# Does selective digestive decontamination (SDD) increase antibiotic resistance? Long-term comparison of two intensive care units (with and without SDD) of the same tertiary hospital

**DOI:** 10.1007/s10096-024-04792-0

**Published:** 2024-03-09

**Authors:** Alicia Rodríguez-Gascón, Yanire Lloréns-Villar, María Ángeles Solinís, Helena Barrasa, Andrés Canut-Blasco

**Affiliations:** 1 Pharmacokinetic, Nanotechnology and Gene Therapy Group (Pharma Nano Gene), Faculty of Pharmacy, Centro de Investigación Lascaray Ikergunea, University of the Basque Country UPV/EHU, Paseo de la Universidad 7, Vitoria-Gasteiz, 01006 Spain; 2 Bioaraba, Microbiology, Infectious Disease, Antimicrobial Agents, and Gene Therapy, Vitoria-Gasteiz, 01009 Spain; 3 Hospital Pharmacy Service, Araba University Hospital, Osakidetza Basque Health Service, Vitoria-Gasteiz, 01009 Spain; 4 Intensive Care Unit, Osakidetza Basque Health Service, Araba University Hospital, Vitoria-Gasteiz, 01009 Spain; 5 Microbiology Service, Osakidetza Basque Health Service, Araba University Hospital, Vitoria-Gasteiz, 01009 Spain

**Keywords:** Selective digestive decontamination, Critically ill patients, Antimicrobial resistance, Antimicrobial consumption

## Abstract

**Purpose:**

The aim of this study was to to compare the antimicrobial resistance rate and its relationship with the antibiotic consumption in two separate Intensive Care Units (ICUs) of the same hospital, one with and other without selective decontamination of the digestive tract (SDD).

**Methods:**

We performed a retrospective study in the two ICUs of the Araba University Hospital. Trauma and neurosurgical patients are admitted to the SDD-ICU, and general digestive surgery patients go to the no SDD-ICU. From 2014 to 2018 we analyzed the number of isolates, and the bacterial resistance trends of 47 antimicrobial-microorganism combinations. Additionally, antimicrobial consumption was estimated in both ICUs. Resistance rates were also compared with those reported in ENVIN-HELICS Spanish national registry.

**Results:**

In the ICU with SDD protocol, there was a significant decrease in the resistance of *E. coli* to amoxicillin/clavulanic acid and in the resistance of *E. faecalis* to high concentration of gentamycin and high concentration of streptomycin. A significant increase of resistance of *Staphylococcus* coagulasa negative (CoNS) to linezolid in the no SDD-ICU was also detected. Overall, the level of resistance in the SDD-ICU was lower or of the same order than in the ICU without SDD and that reported in the Spanish national registry.

**Conclusions:**

SDD had neither a clinically relevant impact on emergence and spread of resistance, nor in the overall systemic antimicrobial use. The patient type rather than the SDD protocol showed to condition the ecology and therefore, the resistance rate in the ICUs.

**Supplementary Information:**

The online version contains supplementary material available at 10.1007/s10096-024-04792-0.

## Introduction

Selective decontamination of the digestive tract (SDD), proposed almost 40 years ago, is a measure to prevent infection in intensive care unit (ICU) patients [1]. It involves the application of topical, nonabsorbable antimicrobial agents (usually colistin, tobramycin, and nystatin) that selectively spare the anaerobic flora. Apart from the topical prophylaxis applied to the patients, intravenous antibiotics, usually a second- or third-generation cephalosporin (without activity against the anaerobic Gram-negative microbiota) is added to treat any incubating infection caused by the commensal flora of the respiratory tract at the time of hospital admission [2]. The control of gut overgrowth is one mechanism proposed to explain how SDD regimens might prevent ICU-acquired infection; therefore the aim of SDD is to eradicate colonization with aerobic Gram-negative bacteria, *Staphylococcus aureus* and yeasts, while leaving the anaerobic flora intact (and thereby improve patient outcomes) [3]. Patients with an expected ICU stay of at least two or three days, and receiving mechanical ventilation are the targeted population, and immediately upon ICU admission the preferred moment to start the SDD protocol [4, 5]. This intervention has been extensively studied and has been proven to be effective in infection prevention and mortality reduction, particularly in settings with a low prevalence of multidrug-resistant bacteria [6–8].

In spite of the benefits of SDD, its implementation in the ICUs is still a matter of debate [9–11] mainly due to concerns that it may promote the development of antimicrobial-resistant pathogens [12–14]. In settings with relatively low prevalence of multidrug-resistant pathogens, such as ICUs in the Netherlands, Australia, and New Zealand, evidence that the implementation of SDD has a negative impact in resistance ecology is scarce [2, 15]. In a recent systematic review and meta-analysis in which 32 randomized clinical trials including 24,389 participants were analyzed, the use of SDD in ICU adults treated with mechanical ventilation was associated with lower hospital mortality in comparison with standard care or placebo, but evidence regarding the effect of SDD on antimicrobial resistance was of very low certainty [16]. For settings with high prevalence of multidrug-resistant microorganisms, large-scale studies are still needed to evaluate the effect of SDD on resistance rates.

In a previous study carried out by our group [17], we did not detect relevant differences in the overall susceptibility rate before and after the implementation of an SDD protocol in an ICU of a tertiary hospital of the north of Spain, a geographical area with moderate to high rate of antibiotic resistance. In the present investigation, our aim was to compare the antimicrobial resistance rate and its relationship with the antibiotic consumption in two separate ICUs of the same hospital, one with SDD and other without SDD.

## Materials and methods

The study was carried out in the two ICUs of the Araba University Hospital (Vitoria-Gasteiz, Spain), an 800 beds tertiary care teaching facility. Both ICUs are located in two different buildings, and had different staff. Mainly trauma and neurosurgical patients are admitted to the ICU with SDD, a 13 bed ICU where SDD was implemented in 2002. Mainly general digestive surgery patients go to the ICU without SDD (no SDD-ICU), with 18 beds. In the SDD-ICU, SDD was administered to patients expected to require mechanical ventilation for more than 48 h. A 2% mixture gentamycin (replaced by tobramycin in 2017), colistin and amphotericin B was applied on the buccal mucosa, and a suspension of the same drugs (respective doses of 80, 100 and 500 mg) was provided in the gastrointestinal tract at 6-h intervals. For the first 3 days, systemic ceftriaxone (2 g IV a day) was administered to all SDD patients. The SDD of the digestive tract started on the day of tracheal intubation and continued until the patients were weaned from mechanical ventilation.

The mean age of the patients (63 vs. 66 years-old in the SDD-ICU and no SDD-ICU, respectively), the stay length (5 vs. 4 days in the SDD-ICU and no SDD-ICU, respectively) and the number of admissions/year (880 vs. 1058 in the SDD-ICU and no SDD-ICU, respectively) were similar in the two ICUs.

Except the SDD, the protocols for infection control were the same in the two ICUs; among others International Standard ISO 9001:2000 guidance, the ‘Bacteremia Zero’ program, the ‘Zero-VAP’ (where VAP stands for ventilator-associated pneumonia) bundle, and Zero Resistance program.

This study met the exemption criteria of the ethics committee of clinical research because the data analyzed were collected in the routine practice and did not allow the identification of patients.

### Bacterial isolates and antimicrobial resistance

We retrospectively collected the number of isolates, resistance rates and bacterial resistance trends in 47 antimicrobial-microorganism combinations in both ICUs from 2014 to 2018 (Table S1 supplementary material). Quarterly data of isolates and resistance rates were used (in the SDD-ICU, data collection started from the first quarter of 2014, and in the no SDD-ICU, data collection started from the last quarter of 2014). For enterococci, we also tested the high-level aminoglycoside resistance (isolates with gentamycin MIC ≥ 500 mg/L or streptomycin MIC ≥ 1000 mg/L) since it suppresses the synergic effect with cell wall synthesis inhibitors, which compromises the treatment in case of enterococcal endocarditis and meningitis. For resistance rate calculations, we considered only the first isolate from the patients admitted to the ICU, according to the CLSI guidelines [18, 19] and the National Guideline [20]. The resistance data, derived from microbiological surveillance and clinical care were re-interpreted using interpretive criteria current at the time of analysis and based on the Clinical and Laboratory Standards Institute (CLSI) [19]. Data. Surveillance samples from the nasal, throat and rectum (swabs), were obtained twice weekly. Blood, urine and another diagnostic samples were taken on clinical indication only. The laboratory data from the Microbiology department were managed with Whonet [21], a Windows-based database software package for the management of microbiology laboratory data and the analysis of antimicrobial susceptibility test results. It is a free software developed by the WHO Collaborating Centre for Surveillance of Antimicrobial Resistance for laboratory-based surveillance of infectious diseases and antimicrobial resistance.

We also obtained data of antimicrobial resistance from the ENVIN-HELICS national registry [22] (Spanish Surveillance Study of Nosocomial Infection in the ICU, which includes data from all patients admitted to the participating ICUs for more than 24 h). According to the ENVIN-HELICS protocol, for each pathogen, only the first isolate is included, even if there are several infections or colonizations. The source that assumes the greatest severity (for example, blood over abdominal drainage) is chosen. For this work, data from the 2018 report were used (corresponding to resistance data from April 1 to June 30, 2018), which includes a total of 27.514 patients and 219 ICUs. Less than 5% of these ICUs have implanted the SDD; therefore, these data were used as reference resistance values in absence SDD.

### Antibiotic use

From 2014 to 2018, quarterly quantities of the antimicrobial drugs consumed in each ICU were retrospectively collected from the pharmacy stock management system (in both ICUs, data collection started in the first quarter of 2014). Similarly, numbers of occupied bed-days (OBD) per quarter were obtained from the hospital´s admission department. Antibacterial consumption, expressed as DDDs/100 patient-days, was calculated quarterly in each ICU, according to the 2020 version of the ATC/DDD classification [23]. Prophylactic and therapeutic medication were not distinguished. For every antibiotic, the amount prescribed was also expressed as the percentage of the total antibiotics prescribed.

### Statistical analysis

Trends in the rate of resistance and in the antimicrobial consumption were analyzed with linear correlation. A p value of < 0.05 was considered statistically significant. Statistical analyses were performed with the IBM® SPSS® software, (Statistics for Windows, Version 27). When resistance was always 0, the resistance trends was not evaluated.

## Results

**Fig. 1:**
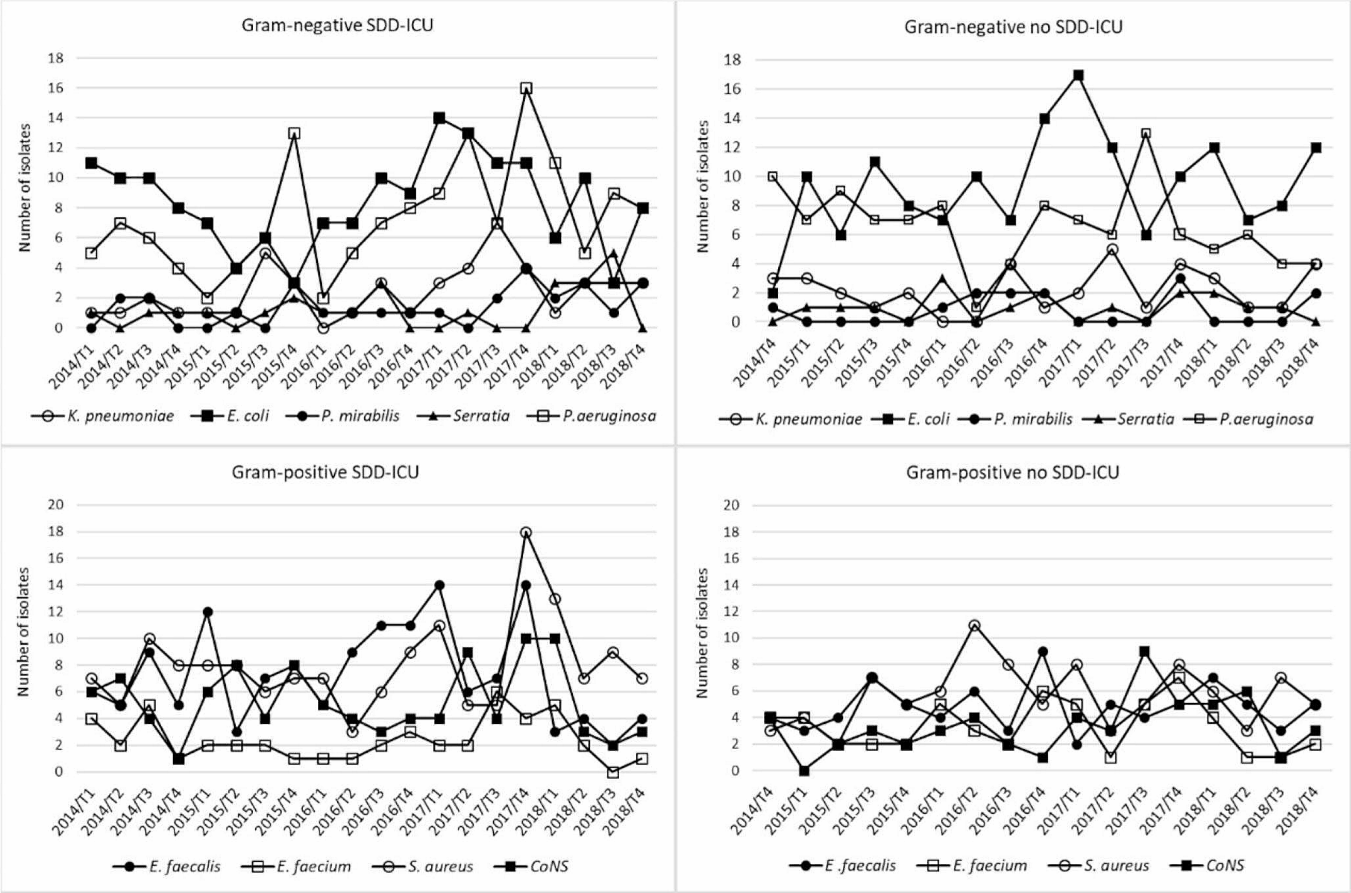
Number of gram-negative and gram-positive isolates in the two ICUs of the HUA from 2014 to 2018

Figure 1 features the evolution of the number of Gram-positive and Gram-negative isolates in the two ICUs from 2014 to 2018. The more frequent Gram-negative microorganisms were *P. aeruginosa* and *E. coli*, and no relevant differences were found between the two UCIs. Overall, the number of Gram-positive isolates were higher in the SDD-ICU than in the no SDD-ICU.

In both ICUs, the most prescribed antibiotics (apart from the antibiotics included in the SDD protocol in the SDD-ICU) were piperacillin/tazobactam, amoxicillin/clavulanic acid, cloxacillin, ceftriaxone, meropenem, levofloxacin, and linezolid (Supplementary Fig. 1). Regarding the consumption rate of each antibiotic with respect to the total antibiotics prescribed in each ICU (Supplementary Table 2), the greater differences between the two ICUs were for cloxacillin (9.51% in the SDD-ICU vs. 3.62% in the no SDD-ICU), cefepime (5.28% in the SDD-ICU vs. 2.50% in the no SDD-ICU), piperacillin/tazobactam (8.81% in the SDD-ICU vs. 19.20% in the no SDD-ICU), and levofloxacin (6.68% in the SDD-ICU vs. 11.08% in the no SDD-ICU).

Table 1 shows the antimicrobial consumption trends in the two ICUs from 2014 to 2018 (only significant trends are included). In the SDD-ICU, the consumption of amikacin, gentamycin and piperacillin/tazobactam significantly decreased, whereas the consumption of ceftazidime, tobramycin, and all aminoglycosides significantly increased. In the no SDD-ICU, the use of aztreonam, daptomycin, linezolid, meropenem, all beta-lactams, and all antimicrobials significantly increased.

**Table 1:** Significant antimicrobial consumption (DDDs/100 patients-days) trends in the two ICUs from 2014 to 2018

	SDD-ICU				NO SDD-ICU				
Antimicrobial	Slope (95% CI)	p value	R	Trend	Slope (95% CI)	p value	R	Trend	
Amikacin	-0.20 (-0.39 to -0.17)	0.034	0.476	↓					
Aztreonam					0.05 (0.003 to 0.10)	0.038	0.465	↑	
Ceftazidime	0.32 (0.09 to 0.55)	0.009	0.566	↑					
Daptomycin					0.23 (0.05 to 0.42)	0.017	0.527	↑	
Gentamycin	-4.10 (-6.17 to -2.03)	< 0.001	0.700	↓					
Linezolid					0.03 (0.10 to 0.54)	0.007	0.583	↑	
Meropenem					0.61 (0.19 to 1.02)	0.006	0.533	↑	
Piperacillin/tazobactam	-0.37 (-0.69 to -0.25)	0.025	0.500	↓					
Tobramycin	7.36 (4.35 to 10.38)	< 0.001	0.771	↑		-	-		
All beta-lactams					1.52 (0.67 to 2.37)	0.001	0.663	↑	
All aminoglycosides	3.06 (1.29 to 4.82)	0.002	0.651	↑		-	-		
All antimicrobials*					2.05 (0.86 to 3.24)	0.002	0.648	↑	

* In all antimicrobial agents, we included those used in the ICU except the gentamycin, tobramycin and colistin used in the SDD protocol. R: correlation coefficient. CI: confident interval

Table 2 shows significant antibiotic resistance trends from 2014 to 2018 in the two ICUs. In the SDD-ICU, there was a significant decrease in the resistance of *E. coli* to amoxicillin/clavulanic acid and in the resistance of *E. faecalis* to high concentration of gentamycin and high concentration of streptomycin. In the no SDD-ICU, we detected a significant increase of the resistance of *K. pneumoniae* to ceftazidime and cefotaxime, and the resistance of CoNS to linezolid.

**Table 2:** Resistance trends of isolates from 2014 to 2018 in the two ICUs

Microorganism	Antimicrobial agent	Mean ± SD	Slope (95% CI)	p value	R	Trend	N
**SDD-ICU**							
*Escherichia* *coli*	Amoxicillin/clavulanic acid	27 ± 22	-1.84 (-3.43 to -0.25)	0.025	-0.498	↓	143
*Enterococcus faecalis*	High concentration of gentamycin (500 mg/L)	66 ± 22	-1.79 (-3.45 to -0.14)	0.035	-0.473	↓	139
	High concentration of streptomycin (1000 mg/L)	55 ± 21	-1.90 (-3.43 to -0.36)	0.018	-0.522	↓	139
**No SDD-ICU**							
*Klebsiella pneumoniae*	Cefotaxime	15 ± 31	3.31 (0.50 to 6.11)	0.018	0.564	↑	37
	Ceftazidime	15 ± 31	3.31 (0.50 to 6.11)	0.018	0.564	↑	37
CoNS	Linezolid	19 ± 28	3.34 (1.08 to 5.60)	0.007	0.610	↑	58

CoNS: coagulase-negative staphylococci. CI: confident interval. N: number or isolates. R: correlation coefficient. SD: standard deviation

Table 3 presents a comparison of the resistance rate of the most frequent microorganisms in the SDD-ICU and no SDD-ICU in 2018 (annual data) and those published in the ENVIN-HELICS report for 2018. Except for ceftazidime and piperacillin/tazobactam, resistance rate of *E. coli* in the no SDD-ICU were comparable to that from the ENVIN-HELICS study, and higher than those in the SDD-ICU. Regarding *P. aeruginosa*, the higher difference was detected for amikacin, ceftazidime, cefepime and piperacillin/tazobactam (higher in ENVIN-HELICS), and for colistin and imipenem, (higher in our two ICUs). Resistance rate of *S. aureus* to oxacillin was lower in the SDD-ICU than in the no SDD-ICU, and comparable to the ENVIN-HELICS report. In both ICUs and in the national survey, resistance of CoNS to linezolid was of the same order.

**Table 3:** Resistance rates (% of isolates) of the most frequent microorganisms from the two ICUs (annual data of 2018) compared with data from the ENVIN-HELICS 2018 report [22]

Microorganism	Antimicrobial agent				SDD-ICU		No SDD-ICU		ENVIN-HELICS	
**Gram-negative organisms**										
***Escherichia coli***	Amoxicillin/clavulanic acid				6		19		27	
	Cefepime				6		15		20	
	Cefotaxime				8		21		13	
	Ceftazidime				6		5		13	
	Ciprofloxacin				25		40		40	
	Gentamycin				13		17		10	
	Levofloxacin				24		41		43	
	Piperacillin/tazobactam				3		2		14	
***Pseudomonas aeruginosa***	Amikacin				2		0		17	
	Cefepime				3		6		33	
	Ceftazidime				11		15		30	
	Ciprofloxacin				35		56		35	
	Colistin				21		13		7	
	Imipenem				38		56		33	
	Levofloxacin				38		58		41	
	Meropenem				20		36		31	
	Piperacillin/tazobactam				11		15		33	
	Tobramycin				21		40		NA	
	Aztreonam				9		20		NA	
**Gram-positive organisms**										
***Enterococcus faecalis***	High concentration of gentamycin (500 mg/L)				54		14		NA	
	High concentration of streptomycin (1000 mg/L)				42		7		NA	
	Vancomycin				2		0		0	
	Linezolid				0		0		3	
***Staphylococcus aureus***	Oxacillin				17		32		14	
	Vancomycin				0		0		0	
	Daptomycin				0		0		0	
	Linezolid				0		0		0	
**CoNS**	Oxacillin				69		79		89	
	Vancomycin				0		0		1	
	Daptomycin				0		0		2	
	Linezolid				13		12		18	

CoNS: coagulase-negative staphylococci. NA: not available

## Discussion

The effect of SDD on ICU-level antimicrobial resistance rates is largely underexplored and existing studies of selective decontamination have not answered the question of how selective decontamination affects ICU-level antimicrobial resistance rates over time [2]. One of the difficulties to evaluate the effect of SDD on the emergence of resistance rate is the controversy due to variable study design including control groups that may not receive SDD protocol. In this observational and retrospective study, we have compared the resistance rate in two ICUs belonging to the same institution, one with SDD protocol and the other without it. According to admission criteria, trauma and neurosurgical patients with ventriculoperitoneal shunts are admitted to SDD-ICU. It is important to note that trauma patients at the ICU often require prolonged mechanical ventilation, and therefore, they may benefit from SDD. On the contrary, general digestive surgery patients are admitted to the no SDD-ICU, where no SDD has been implemented.

The application or not of SDD protocols may conditions the circulating microorganisms at the ICU [1]. In fact, the antimicrobial spectrum of the classic SDD regimen lacks coverage of most Gram-positive bacteria, and increased rates of colonization and infection with enterococci and methicillin-resistant *S. aureus* (MRSA) can be expected as it has been reported [24, 25]. In line with this, we detected a higher number of *E. faecalis* and *S. aureus* isolates in the ICU with the SDD protocol, and a decrease in the number of *E. faecium* isolates. These results are in agreement with those found by van der Bij et al. [26] in Dutch ICUs with and without SDD, who reported that the introduction of SOD (selective oropharyngeal decontamination)/SDD was associated with increased rates of *S. aureus* and *E. faecalis* isolates in respiratory tract specimens; moreover, they observed an increase in *E. faecium* isolates in the absence of SOD/SDD.

The differences in the prescription of some antimicrobials between the two ICUs may be justified in part by the patient and infection type, and in part by the application of the SDD protocol. Directly related to the SDD protocol, the consumption of gentamycin, tobramycin and colistin was much higher in the SDD-ICU than in the no SDD-ICU, where these antibiotics are hardly prescribed. The change in the SDD protocol in 2017 (gentamycin was replaced by tobramycin) explains the significant increase of tobramycin consumption and the significant decrease of gentamycin consumption in the SDD-ICU. Our SDD protocol included ceftriaxone since resistance rate of *Enterobacterales* is below 10%. In other countries with moderate-to-high-prevalence of antibiotic resistant bacteria, including third-generation cephalosporin-resistant *Enterobacterales*, intravenous cephalosporin is absent in the SDD protocols [5] since such prophylaxis was considered unappropriated.

Regarding the kind of patient and infection, and according to the admission criteria, gastrointestinal surgery patients go to no SDD-ICU, and since these patients are at high risk of abdominal infection, the consumption of antibiotics indicated for this kind of infections are higher in this ICU, such us piperacillin/tazobactam and levofloxacin. On the contrary, infections by Gram-positive microorganisms are more frequent in SDD-ICU, who admit the trauma and neurosurgical patients, and therefore, antibiotics such as cloxacillin and cephalosporins, were more prescribed in this ICU.

The high meropenem prescription in our ICUs may be related to the high prevalence of multiresistent *P. aeruginosa* in our region (north of Spain), as demonstrated in a large-scale Spanish nationwide survey of *P. aeruginosa* infections [27]. This pathogen has remarkable capacity for acquiring new resistance mechanisms under selective antibiotic pressure [28, 29]; in fact, carbapenem-resistant *P. aeruginosa* has become common worldwide, and it is known that resistance is associated with carbapenems use.

The increase along the period of study of linezolid and daptomycin consumption, both antibiotics indicated for the treatment of Gram-positive microorganism infections, more frequent in this ICU, may be due to the introduction of the generic drug products in the therapeutic guide of the hospital, linezolid in 2015 and daptomycin in 2018. The increase of prescription of these antibiotics after generic approval is also reported in other studies [30]. In a recent study [6], overall total daily defined doses for each antibiotic class were not significantly different between SDD and standard of care. In our study, we detected a significant increase of all beta-lactams and all antibiotics use over time in the ICU without SDD, while in the ICU with SDD, the consumption of both, all beta-lactams and all antibiotics remained constant. These results are in agreement with Daneman [31], which postulates that the use of prophylactic selective decontamination could even lead to reductions in the need for therapeutic antimicrobials.

We found reduction in the resistance rate of *E. coli* to amoxicillin/clavulanic acid in the SDD-ICU, which was not detected in the no SDD-ICU. In a previous study [32], it was shown that previous exposition to piperacillin/tazobactam is a predisposing factor for amoxicillin/clavulanic acid-resistant *E. coli* isolates. In this sense, the reduction of the resistance of *E. coli* to amoxicillin/clavulanic acid in the SDD-ICU could be related to the significant reduction of the piperacillin/tazobactam consumption in this ICU. Additionally, significant decrease of *E. faecalis* resistant to high concentration of gentamycin and to high concentration of streptomycin was also found, which is probably related to the change of gentamycin by tobramycin the SDD protocol. *K. pneumoniae* is a major pathogen implicated in nosocomial infections that is known to spread easily, and it is frequently associated with resistance to the highest-priority critically important antimicrobial agents [33]. We found a significant increase of *K. pneumoniae* to cefotaxime and ceftazidime in the no SDD-ICU, not observed in the SDD-ICU. This result may be explained, in part, by the higher use of fluoroquinolones (mainly levofloxacin) in this ICU, since the consumption of these antibiotics is associated with the development of extended-spectrum-beta-lactamases (ESBL) [34], one of the most common resistance mechanisms of *K. pneumoniae* [35, 36]. This resistance pattern may be also explained by the decontamination protocol, since SDD has demonstrated to be efficacious in controlling colonization with ESBL-producing bacteria [37]. However, considering the low number of isolates, this result must be interpreted with caution.

Resistance of CoNS to linezolid increased in the ICU without SDD protocol, where a significant increase of this antibiotic use was also found. CoNS are the main pathogens in health care-associated ventriculitis and meningitis (HCAVM) [38]. Although linezolid is not registered for the treatment of CoNS infections, it is widely used off-label for the treatment of meningitis, ventriculitis, osteomyelitis and prosthetic-joint infections caused by CoNS promoting emergence of resistance [39, 40].

Our results in the ICU with SDD are in line with a previous case-control study of 5034 mechanically ventilated critically ill patients over 5-year period, which revealed that ESBL-producing *K. penumoniae* was more common in the ICU patients not treated with SOD [41]. However, contrary to this study, we did not detected increase of vancomycin-resistant *E. faecium* in any of the two ICUs.

In order to better evaluate if the SDD could have an impact on the antimicrobial resistance, we compared the resistance rates of the most frequent microorganisms in the two ICUs of our hospital with those reported in the ENVIN-HELICS national registry [22], which includes data from 219 Spanish ICUs (SDD represents less than 5%). We have confirmed the high resistance level of *P. aeruginosa* against meropenem in our ICUs, similar to the media of Spanish ICUs, which may be related to the high prevalence in our geographical area of the sequence type (ST) 175 [27], as mentioned above. Resistance of *P. aeruginosa* against piperacillin/tazobactam in our ICUs was much lower than that reported in the national survey. In the SDD-ICU, MRSA rate was similar to that reported in the national registry, being lower than in the ICU without SDD; this result may be explained, at least in part, by the contribution of SDD to control the resistance of MRSA. The worrying resistance of CoNS to linezolid observed in our ICUs and in the national registry may be related to the ability of these bacteria to develop resistance quite easily following linezolid exposure [42]. Actually, the rate of linezolid-resistant CoNS is increasing worldwide [43].

Our study presents some limitations. First, since the admission to one or the other ICU is conditioned by the kind of patient, the ICU without SDD protocol cannot strictly be considered as a control. Therefore, in the ICU with SDD, pneumonias are predominantly expected, while abdominal infections may dominate in the no SDD ICU, and accordingly, different pathogens are to be expected and therefore different antibiotics are used. Second, this is a retrospective study including a single hospital, with low number of isolates. However, this study provides unique information on the trends in antibiotic resistance in two ICUs with and without SDD located in a geographical area with medium-high rates of resistance. Third, antibiotic consumption and resistance rate are from pre-Covid 19 period; it has been reported that the occurrence of bacterial pneumonia, mostly ventilator-associated pneumonia (VAP), is a frequent complication in critically ill COVID-19 patients with a higher incidence than in non-COVID-19 patients [44]; moreover, and similarly to other patients admitted to the ICU, in COVID-19 patients, the development of secondary infections prolongs the length of mechanical ventilation and hospitalization, increasing the mortality risk [45]. In a previous study [46], the use of SDD for VAP prevention did not led to changes in the incidence of multidrug-resistant bacteria in COVID-19 patients; therefore, the conclusions obtained in the 2014–2018 period could be applied to the post-Covid 19 age.

In conclusion, by analyzing the resistance rates of the isolates in the two ICUs of our hospital, we have confirmed that SDD had not a clinically relevant impact on emergence and spread of resistance. Additionally, SDD has not been found to increase overall systemic antibiotic use. The patient type rather than the SDD protocol showed to condition the ecology and therefore, the resistance rate at the ICUs.

### Electronic supplementary material

Below is the link to the electronic supplementary material.

Supplementary Material 1

## Data Availability

The datasets generated during and/or analysed during the current study are available from the corresponding author on reasonable request.
