# Supplementary material for: Does selective digestive decontamination (SDD) increase antibiotic resistance? Long-term comparison of two intensive care units (with and without SDD) of the same tertiary hospital
Source: Eur J Clin Microbiol Infect Dis. 2024 Mar 9;43(5):885–93. doi: 10.1007/s10096-024-04792-0 (PMC11108900; doi:10.1007/s10096-024-04792-0)
Supplement: Supplementary file 1 — Supplementary Material 1 [file 10096_2024_4792_MOESM1_ESM.docx]

**Table S1** Antimicrobial-pathogen combinations studied.

| **Gram-negative organisms** | | **Gram-positive organisms** | |
| --- | --- | --- | --- |
| **Microorganism** | **Antimicrobial agent** | **Microorganism** | **Antimicrobial agent** |
| *Escherichia*  *coli* | Amoxicillin/clavulanic acid  Ceftazidime  Cefepime  Cefotaxime  Ciprofloxacin  Gentamycin  Levofloxacin  Piperacillin/tazobactam | *Enterococcus faecalis* | High concentration of gentamycin (500 mg/L)  High concentration of streptomycin (1000 mg/L)  Vancomycin  Linezolid |
| *Klebsiella pneumoniae* | Cefotaxime  Ceftazidime  Meropenem | *Enterococcus faecium* | High concentration of gentamycin (500 mg/L)  High concentration of streptomycin (1000 mg/L) |
| *Proteus*  *mirabilis* | Ceftazidime  Cefepime  Cefotaxime  Ciprofloxacin  Gentamycin  Levofloxacin  Piperacillin/tazobactam | *Staphylococcus aureus* | Oxacillin  Vancomycin  Linezolid |
| *Serratia spp.* | Ceftazidime  Cefepime  Cefotaxime  Ciprofloxacin  Gentamycin  Piperacillin/tazobactam | CoNS | Oxacillin  Vancomicin  Linezolid |
| *Pseudomonas aeruginosa* | Amikacin  Aztreonam  Tobramycin  Cefepime  Ceftazidime  Ciprofloxacin  Levofloxacin  Imipenem  Meropenem  Colistin  Piperacillin/tazobactam |  |  |

CoNS: coagulase-negative staphylococci.

**Table S2** Consumption rate, expressed as percentage of the amount prescribed in relation to the total antibiotic amount (%) in the two ICUs from 2014 to 2018

| **SDD-ICU*** | | **NO SDD-ICU** | |
| --- | --- | --- | --- |
| **Antibiotic** | **(%)** | **Antibiotic** | **(%)** |
| Cloxacillin | 9.51 | Cloxacillin | 3.62 |
| Amoxicillin/clavulanic acid | 9.82 | Amoxicillin/clavulanic acid | 12.43 |
| Cefotaxime | 2.40 | Cefotaxime | 2.00 |
| Ceftazidime | 3.57 | Ceftazidime | 2.26 |
| Cefepime | 5.28 | Cefepime | 2.50 |
| Ceftriaxone | 15.21 | Ceftriaxone | 11.94 |
| Piperacillin/tazobactam | 8.81 | Piperacillin/tazobactam | 19.20 |
| Aztreonam | 0.13 | Aztreonam | 0.41 |
| Imipenem | 0.73 | Imipenem | 0.44 |
| Meropenem | 11.39 | Meropenem | 11.34 |
| **Total beta-lactams** | 66.85 | **Total beta-lactams** | 66.14 |
| Amikacin | 5.24 | Amikacin | 2.79 |
| Gentamycin | 1.02 | Gentamycin | 0.56 |
| Tobramycin | 0.43 | Tobramycin | 0.34 |
| **Total aminoglycosides** | 6.69 | **Total aminoglycosides** | 3.69 |
| Ciprofloxacin | 3.34 | Ciprofloxacin | 2.62 |
| Levofloxacin | 6.68 | Levofloxacin | 11.08 |
| **Total quinolones** | 10.02 | **Total quinolones** | 13.70 |
| Colistin | 0.40 | Colistin | 1.12 |
| Vancomycin | 1.93 | Vancomycin | 3.03 |
| Daptomycin | 4.29 | Daptomycin | 3.85 |
| Linezolid | 9.30 | Linezolid | 8.15 |
| Tigecycline | 0.51 | Tigecycline | 0.31 |
| TOTAL | 100 | TOTAL | 100 |

*Gentamycin, tobramycin and colistin used in the SDD protocol were not included


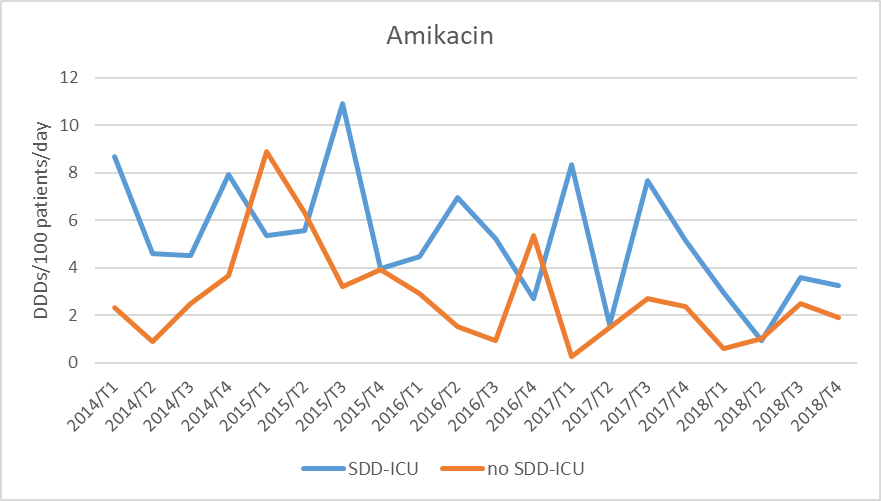

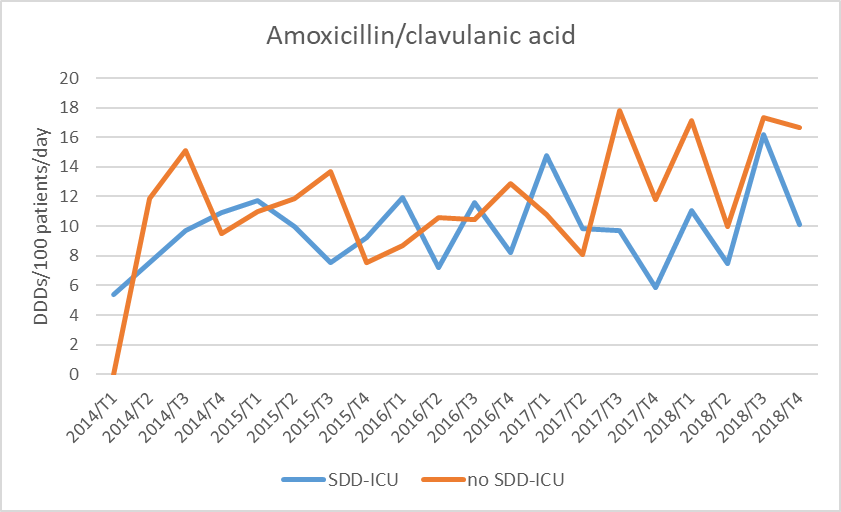

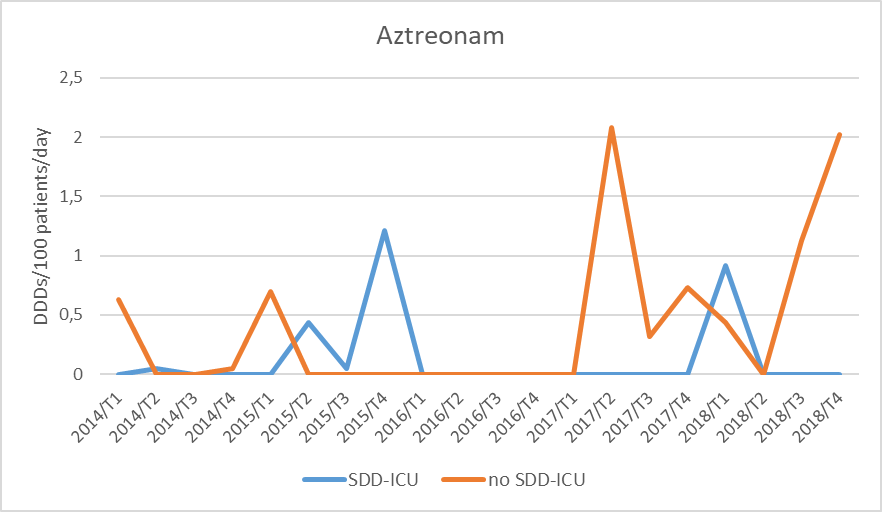

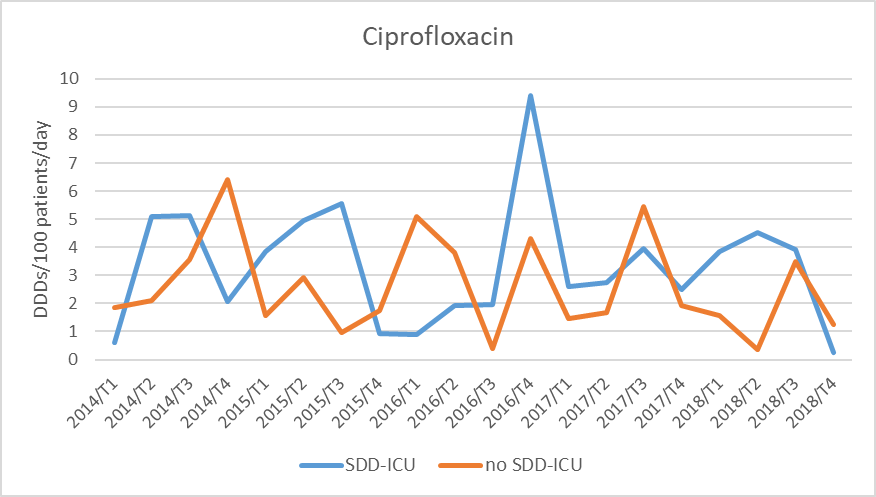

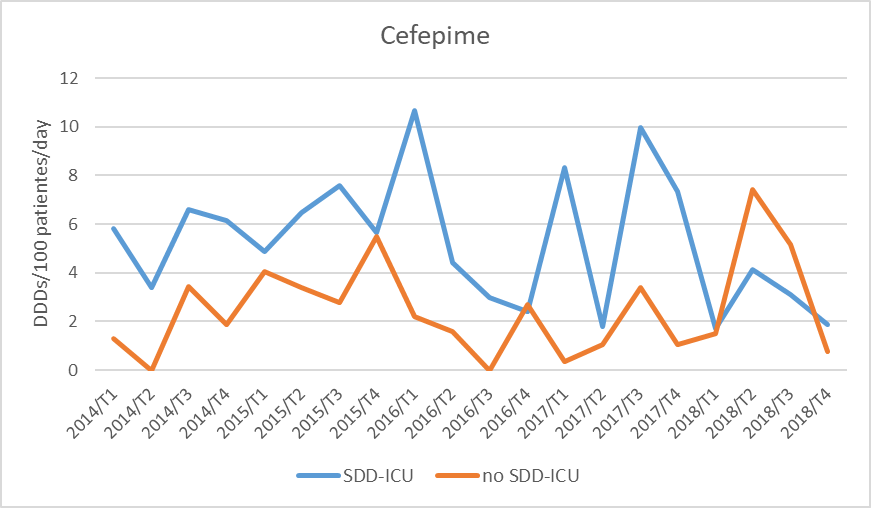

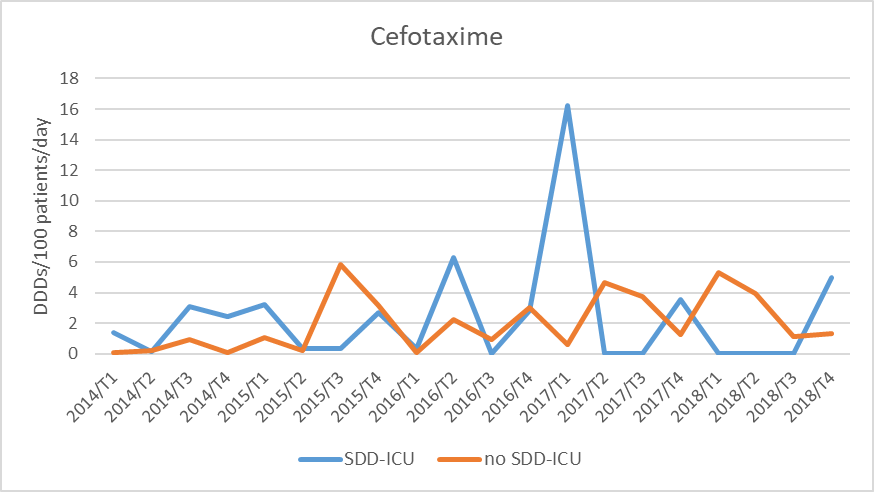

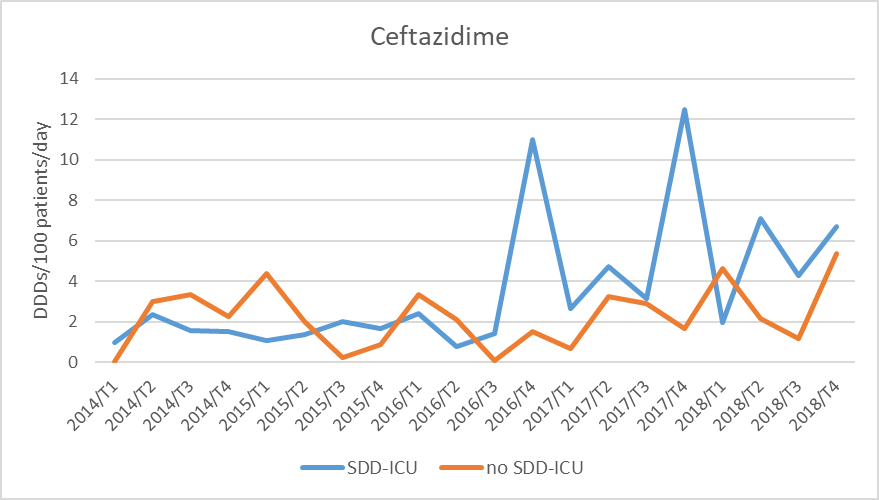

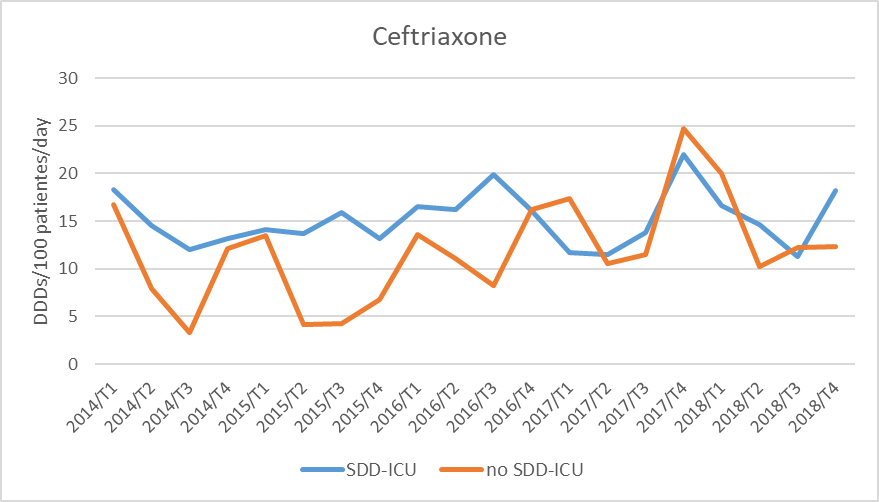

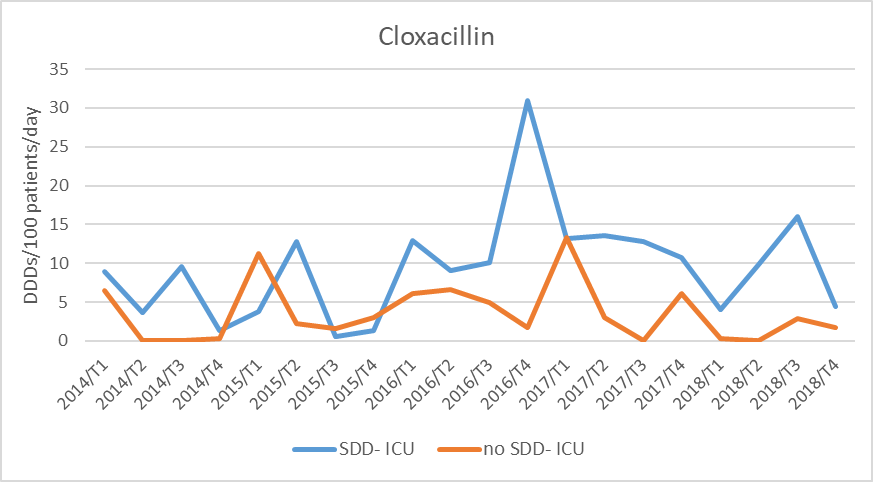

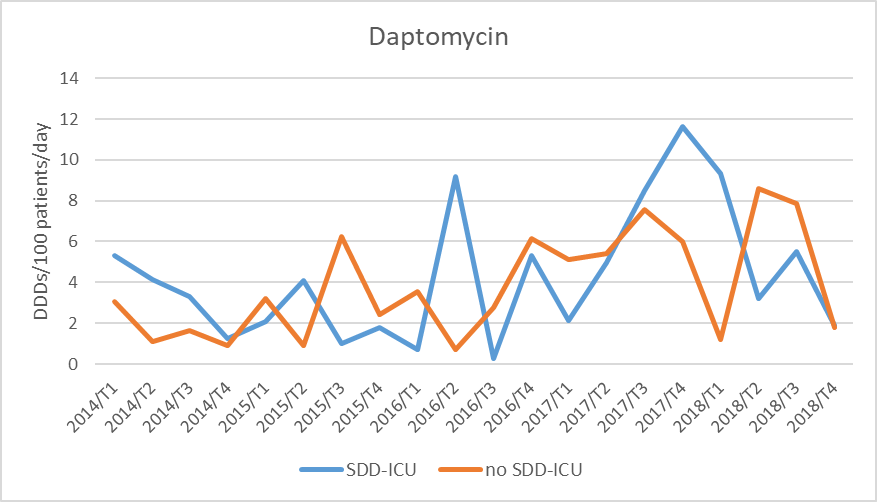


**Figure S1** Quarterly consumption of the antimicrobials used in the ICUs of the HUA (SDD-ICU and no SDD-ICU). In all antibiotics, we included all those used in the ICU except the gentamycin, tobramycin and colistin used in the SDD protocol


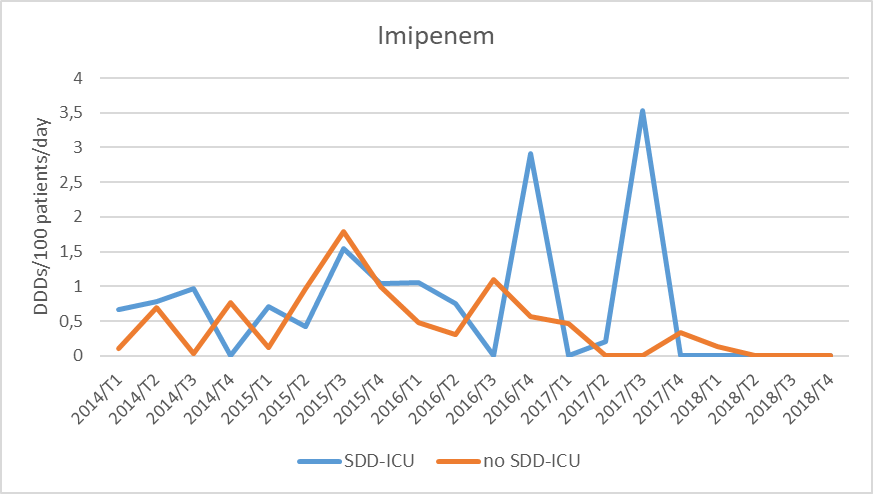

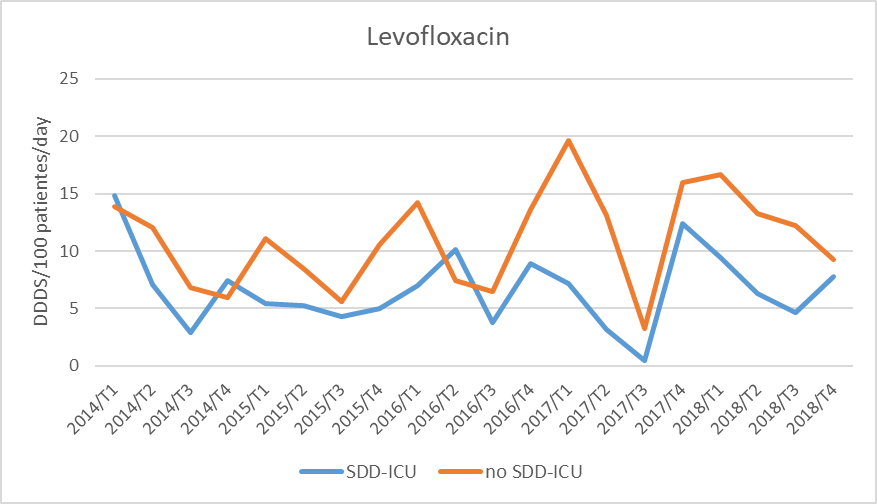

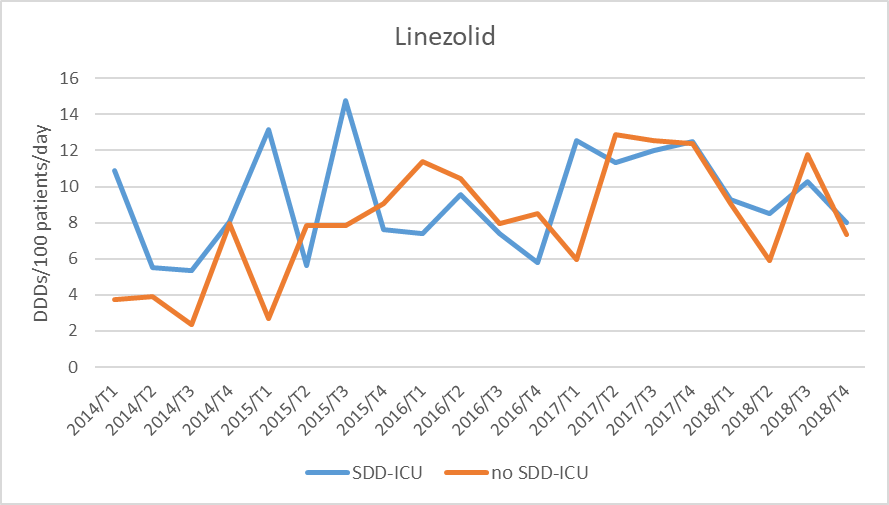

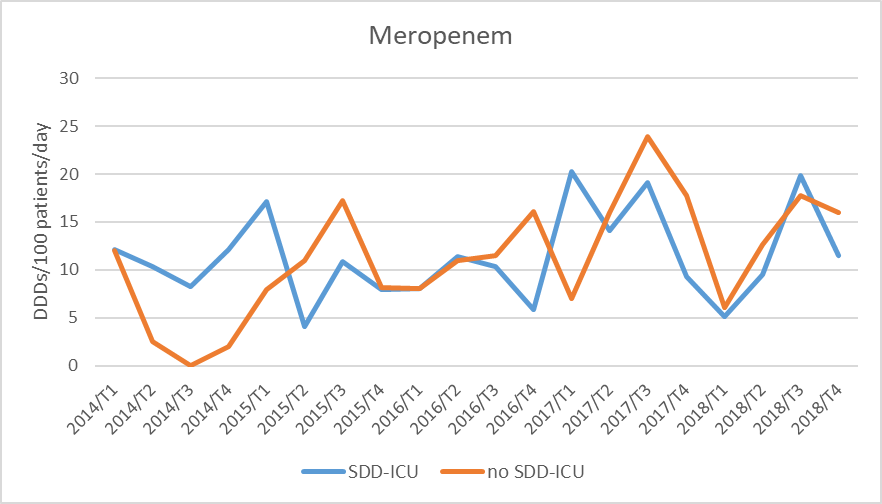

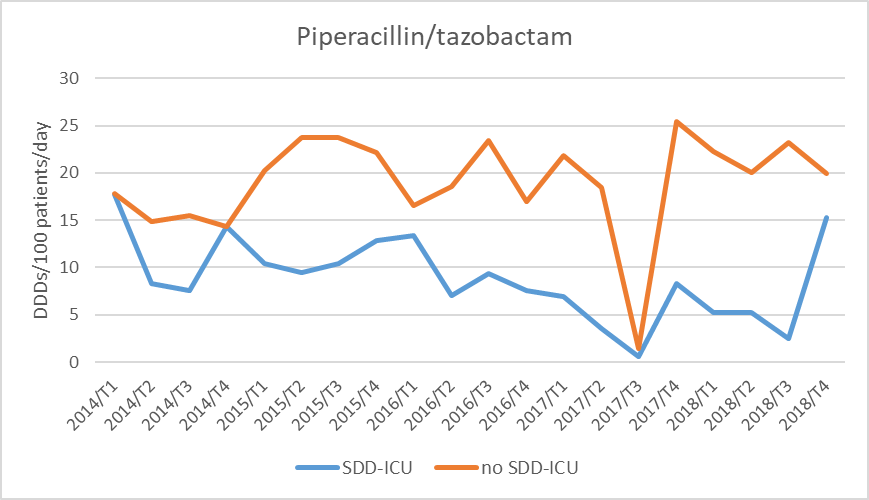

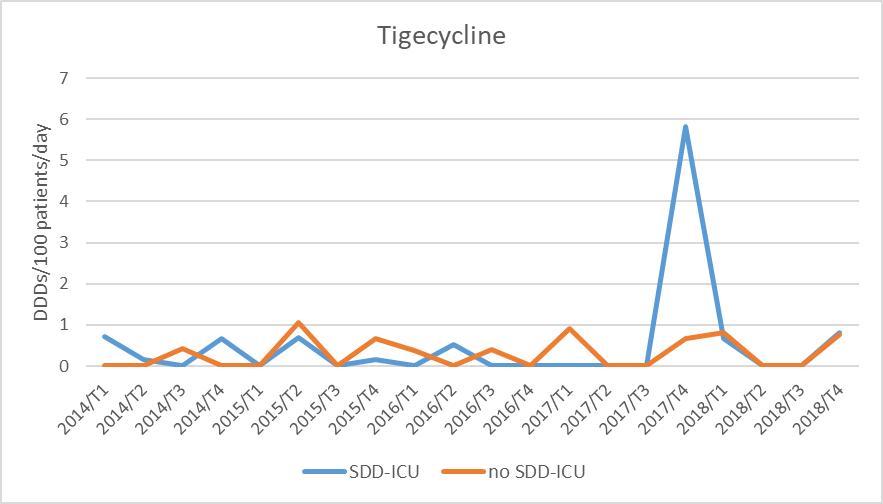

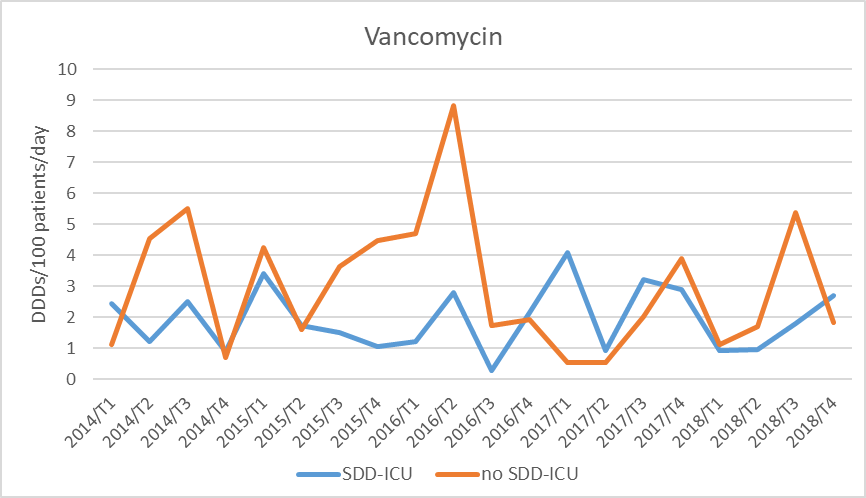


**Figure S1 (cont.)** Quarterly consumption of the antimicrobials used in the ICUs of the HUA (SDD-ICU and no SDD-ICU). In all antibiotics, we included all those used in the ICU except the gentamycin, tobramycin and colistin used in the SDD protocol


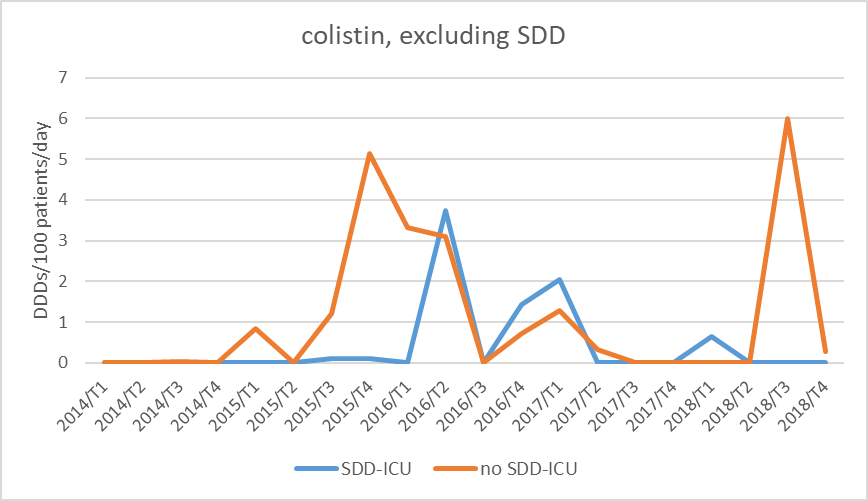

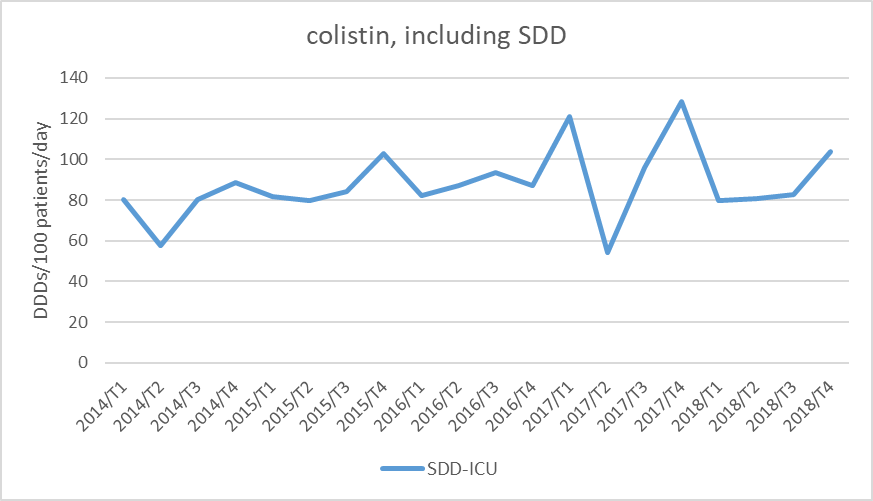

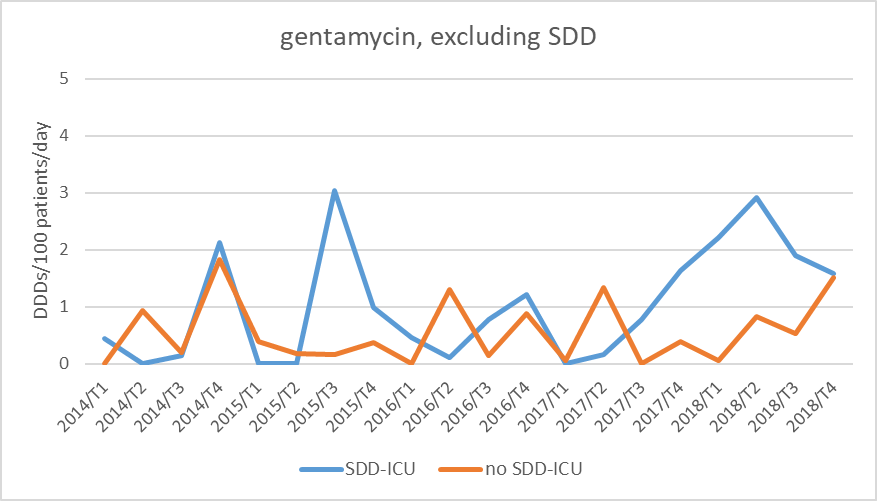

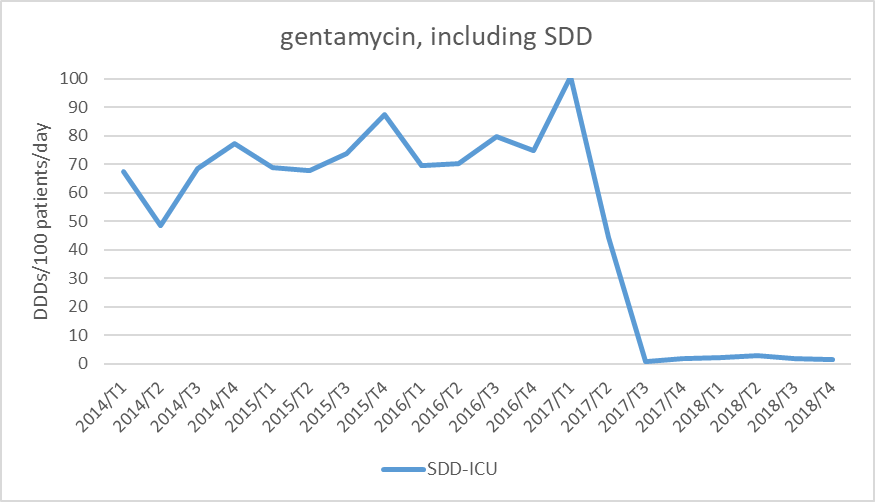

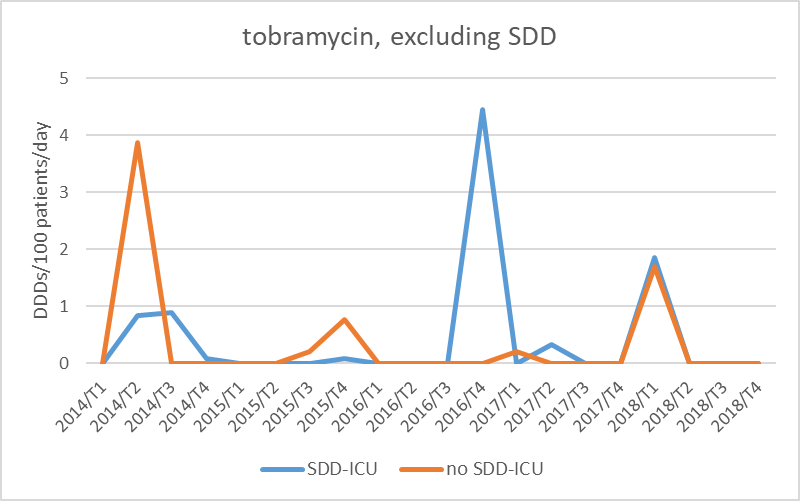

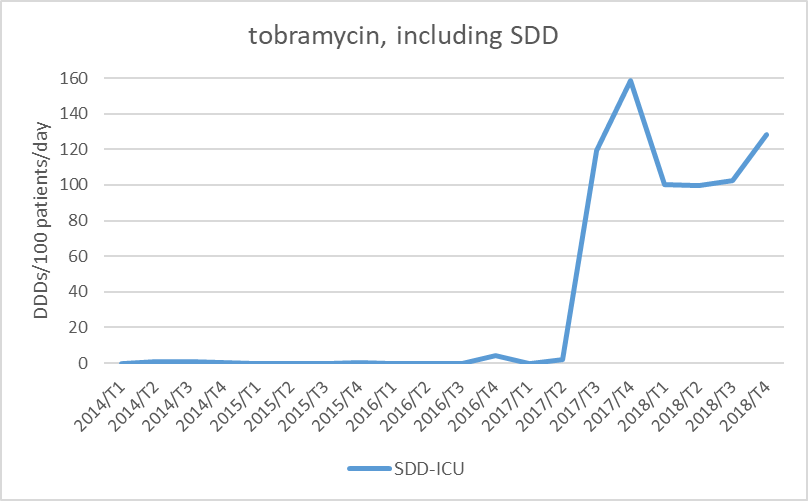

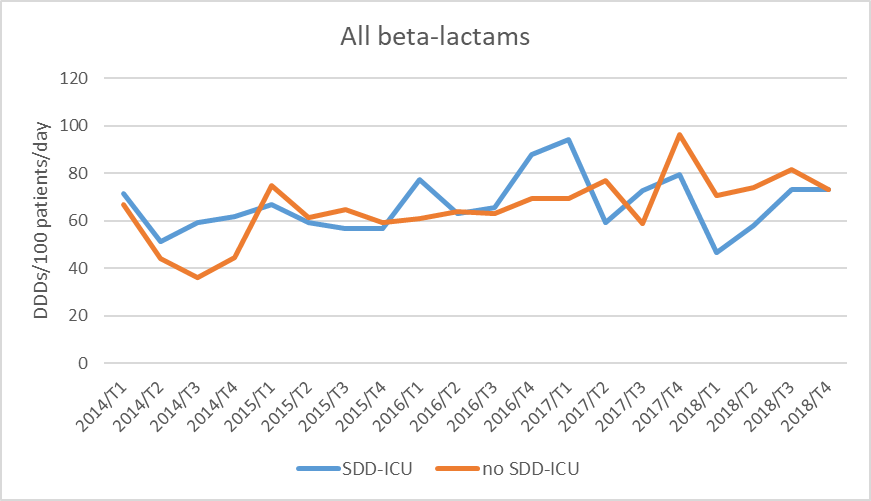

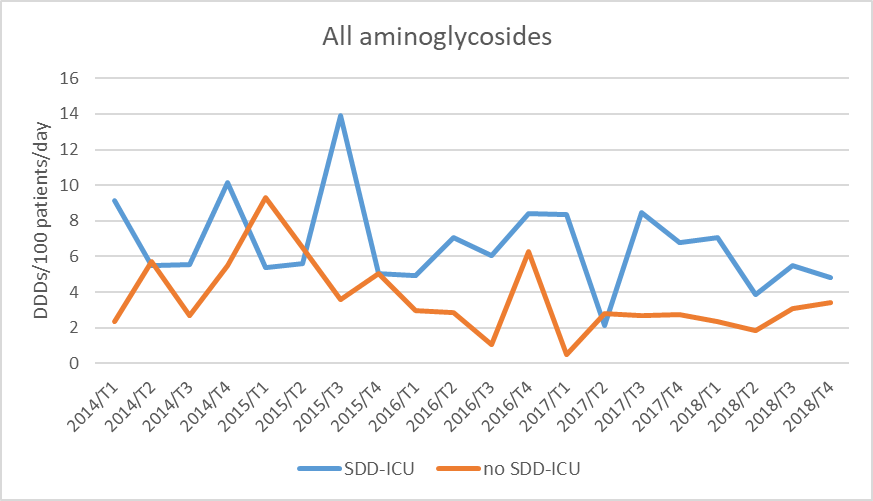

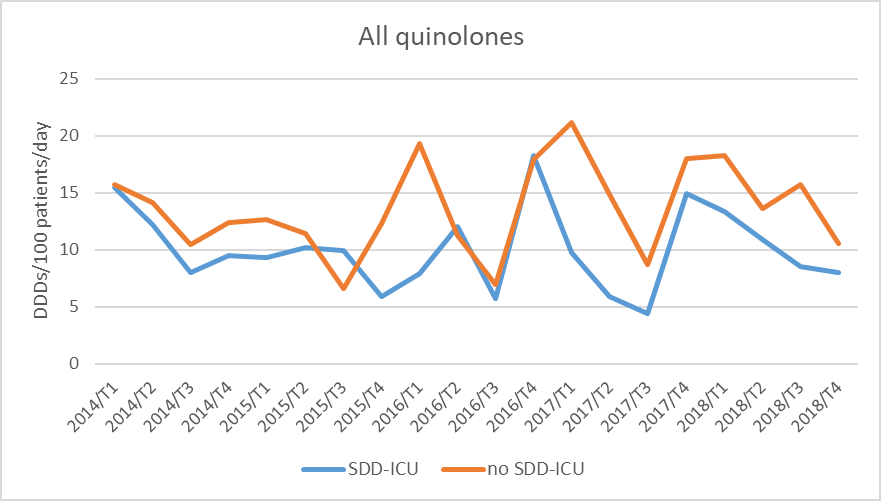
**
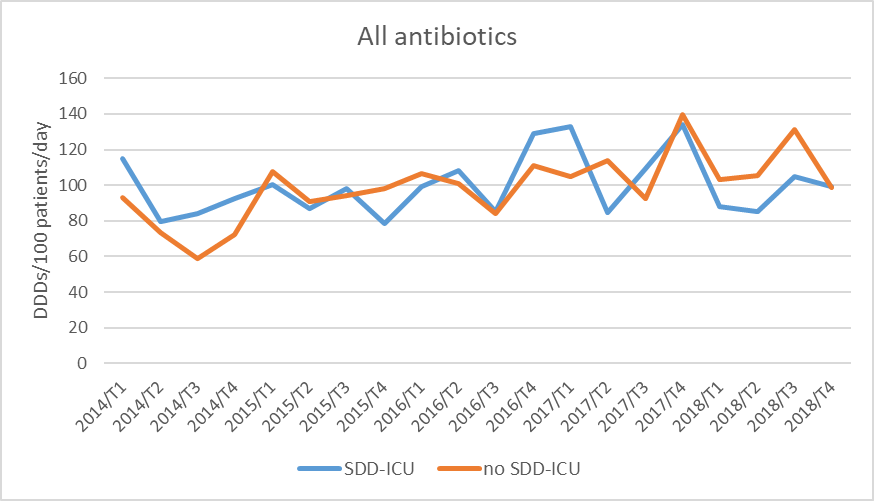
**

**Figure S1 (cont.)** Quarterly consumption of the antimicrobials used in the ICUs of the HUA (SDD-ICU and no SDD-ICU). In all antibiotics, we included all those used in the ICU except the gentamycin, tobramycin and colistin used in the SDD protocol
